# A Comprehensive Review of Medical Imaging Equipment Used in Cadaveric Studies

**DOI:** 10.7759/cureus.2035

**Published:** 2018-01-07

**Authors:** Emily Simonds, Charlotte Wilson, Joe Iwanaga, Tyler Laws, Gary Holley, Rod J Oskouian, R. Shane Tubbs

**Affiliations:** 1 Seattle Science Foundation; 2 Neurosurgery, Swedish Neuroscience Institute; 3 Neurosurgery, Seattle Science Foundation

**Keywords:** cadaver, anatomy, computed tomography, magnetic resonance imaging, endoscopy, ultrasound

## Abstract

Medical imaging techniques have led to great advances in clinical anatomy and forensic pathology. New and emerging technologies allow healthcare professionals to view and understand the human body from different perspectives. This gives way to new and improved interventions, treatment plans, and an overall understanding of the human body. Herein, we present a comprehensive review of the various medical imaging equipment used in cadaveric studies along with their individual strengths and limitations.

## Introduction and background

Numerous studies have been performed on cadavers worldwide. They often require postmortem imaging, which has drastically changed what clinicians know about anatomical variations, how they look at procedures, and how they understand disease progression and treatment. Advanced imaging techniques have helped forensic pathologists determine the cause of death in cases where a conventional autopsy could not be completed [1]. We have reviewed the literature describing imaging techniques and equipment for cadaveric studies.

## Review

Fluoroscopic C-arm

**Figure 1:**
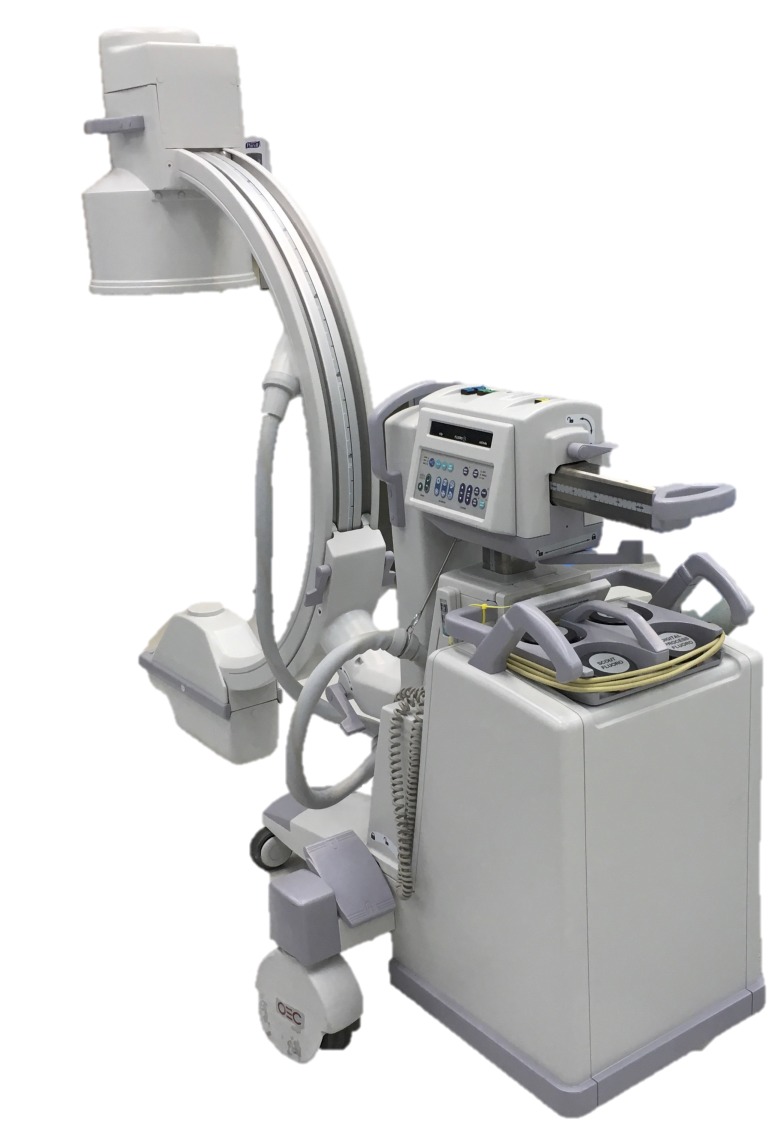
C-Arm

Fluoroscopic C-arms are used in cadaveric studies for determining the placement of orthopedic devices such as screws, new joints, plates, guidewires, and stents, as would be seen in the cardiac catheterization lab (Figure 1) [2]. C-arm is also often used on cadavers to measure the angle, distance, and relationship of screw placements to surrounding structures [3]. The authors cautioned that C-arm and computed tomography (CT) can overestimate the distance of screw placements but measure the angle of the screws accurately. C-arm has also been used to examine the wrists of cadavers and has proved to be more accurate than a multiple-detector computed tomography (MDCT). Although it can produce more artifacts, C-arm is more cost-effective and the image quality is just as good [4]. Its use in cadaveric laboratories is more cost-effective and efficient than other more complex imaging devices and it also occupies significantly less space.

Conventional radiography (X-ray)

X-ray examination is used in many cadaveric studies to document bones before and after manipulation or simulated trauma. X-ray examinations are effective in imaging ribs, femur, and pelvis pre- and post-simulated motor vehicle accidents to examine changes in bone structure for safety assessment. Changes in bone structure were noted and conclusions were drawn regarding safety [5].

X-ray studies were also completed on the ankles of cadavers prior to load testing on the lower legs, then the ankles were re-X-rayed to assess structural changes [6].

X-rays have also been used to measure the cardiopulmonary resuscitation (CPR) force and to look at replacement joint force, and the internal prosthetic forces that can be withstood [7-8].

This is a fairly inexpensive technique that can explore a large area with few artifacts. However, X-ray machines tend to occupy a lot of laboratory space and the positioning of cadavers can be challenging.

Positron emission tomography (PET) scan

PET scans of cadavers have been used to differentiate tissues as well as to examine the effects of different tracers in the brain [9]. PET scans have also been used to test the feasibility of tissue navigation systems in surgical procedures, rather than as a diagnostic tool [10]. A significant amount of Alzheimer’s and Lewy Body dementia work has been conducted on cadavers using PET to determine cortical neuropathology and to compare pre-mortem with post-mortem PET scans directly [9, 11]. Most of this work is comparative in nature as the uptake and metabolism of radioactive compounds in cadavers is very limited. PET scans require specialized rooms and expensive radio-transducing pharmaceuticals. They also demand a significant amount of time. The Canadian Cancer Society estimated that one PET scan cost as much as 2,000 dollars in 2008. This cost is prohibitive and not realistic for cadaveric research.

Computed tomography (CT) scanning

Conventional CT

CT scanning has been used extensively in chest compression postmortems as a teaching tool and to study automatic compression devices for load-distributing band CPR (LDB-CPR) [12]. CT scans have also been used to study bone abnormalities, structure, and potential implant site for bone prosthetics, and hip and knee replacements.

Cone-beam computed tomography (CBCT)

CBCT has been widely used for oral and maxillofacial studies, especially in dentistry. Small foramina in the mandible, maxilla and teeth, and small changes in the hard tissue can be detected by this imaging equipment [13-14].

Micro-computed tomography (micro-CT)

**Figure 2:**
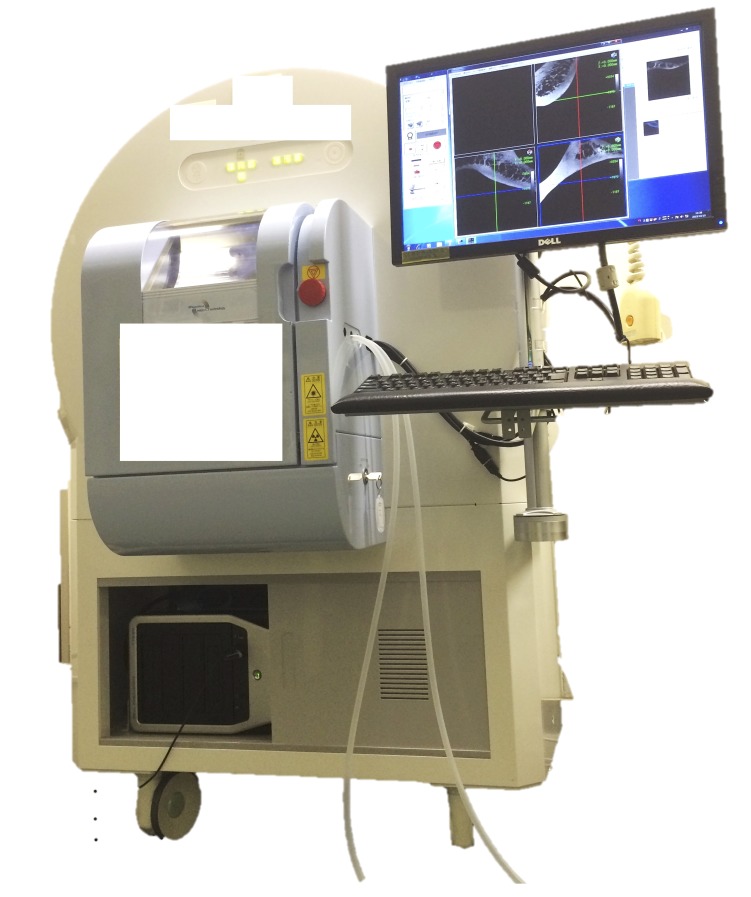
Micro-CT

Micro-CT is an emerging technique being implemented in various fields. In forensic pathology, it allows for exploration of gunshot wounds, marks on bones, bone pathology, age determination, and teeth (Figure 2). It is objective, rapid and relatively inexpensive micro-CT was first used on cadavers to study the mineral density of the alveolar bone and its three-dimensional micro-architecture [15]. The advent of this technology has improved the ability of clinicians to examine the alveolar bone for pathology, to predict whether dental interventions will be successful, and to determine age at the time of death [15-16]. It has also been used to explore smaller joints in cadavers such as the cricothyroid joint (CTJ). In this case, it was used to study the motion of the joint, which had been largely unknown. Anatomists were able to visualize the movement and support the hypothesis that the cricoid cartilage does, in fact, rotate in a visor-like fashion on the inferior cornu of the thyroid cartilage. Micro-CT demonstrated a wobbly-pivoting position that allows for greater mobility and supports the gliding movements of the CTJ [17].

Four-dimensional *(4D)-CT*

4D-CT scans in pediatric cadavers have been used to generate models (phantoms). They have also been used to examine complex joints such as hands and feet. They provide complex detailed analyses of the joint and joint motion [18]. These scans can be completed with contrast, and tend to work better as a postmortem exam to investigate the cause of death due to cardiovascular events. In these cases, the best results are obtained before and after intra-arterial perfusion with a newly-developed barium-containing contrast agent and ventilation of the lungs [19].

Contrasted CT

The use of contrast in cadaver radiology differs from clinical approaches in living patients. Postmortem changes in the vascular system and the absence of blood flow lead to specific problems for postmortem angiography. The images are also challenging to interpret owing to postmortem artifacts that have to be known and are specific to each technique applied. Although the idea of injecting contrast media is old, classic methods cannot simply be extrapolated to modern radiological techniques in forensic medicine, as they are mostly dedicated to single-organ studies or applicable only shortly after death [20]. The degree to which the contrast agent can affect postmortem invasive autopsy studies is not known, so this should be considered prior to injection of any contrast dye.

Unenhanced postmortem CT shows great potential for depicting bones and air-containing structures, but the diagnosis of pathological vascular conditions remains a deficiency of this method because of the lack of adequate techniques for administering contrast medium postmortem [1].

In contrast to adults, postmortem CT yields poor differentiation of visceral tissue in fetuses and children and is of little value except for skeletal injuries and dysplasias [21].

Ultrasound

The vast majority of cadaveric ultrasound studies are not dedicated to diagnosing or visualizing anatomical findings or testing, but in assessing the capabilities of the technique including ultrasound-guided procedures [22]. Also, many ultrasound techniques are used on cadavers for teaching medical professionals and acquiring new procedures.

In lifesaving procedures such as cricothyrotomy, cadaver studies have shown that ultrasound-guided cricothyrotomy has a significantly better outcome in emergency situations where the cricothyroid membrane is likely to be misidentified and the failure can lead to serious complications in a “cannot intubate-cannot oxygenate” situation [23].

Ultrasound has been used to demonstrate that joints such as the knee are more complex than previously thought [24].

Generally speaking, this is a cost- and space-efficient technique for anatomical study but is perhaps underused in cadaver labs.

Doppler ultrasound

Doppler ultrasound has been used to study tendon velocity and displacement for wrist prosthetic devices. Color Doppler technology appears to be particularly useful for the hands. Sonography helps us to understand the behavior of the subsynovial connective tissue during tendon excursion, which can elucidate the role of finger motion [25].

Doppler ultrasound has also been used on cadavers to explore the anatomical feasibility of harvesting an artery for sural artery perforator flap. It is a reliable tool for mapping the arteries of the lower leg for harvesting of the medial and lateral sural artery perforator flap [26].

Three-dimensional (3D) ultrasound

3D ultrasound appears to be more accurate for measuring volume, so it has been used to measure the volumes of various structures such as the kidneys and the upper airway [27-28].

Endoscopy

Endoscopy is used quite extensively on cadavers for surgical and procedural training. In many ways, it provides a window into the body cavities and it often sheds light on anatomical variations. It can also be used in what is called a keyhole autopsy, where an endoscope is deployed to examine anatomy and directly visualize internal organs, with selective tissue biopsy if necessary [29]. It has also been widely used in minimally invasive postmortem autopsies, specifically for obtaining histology and diagnosing gastrointestinal (GI) pathology [30]. Some researchers have used endoscopy to observe small bony canals such as the mandibular canal [31].

Laryngoscopy

Laryngoscopy is an important skill for doctors as it is essential for intubation. Cadavers are implemented to teach the proper technique and to ensure proper intubation and airway management in critical conditions [32]. Further complications, such as upper airway injury and worsening respiratory status, can arise if a laryngoscopy is improperly performed several times. Postmortem autopsies done with endoscopy, on patients with multiple failed intubation attempts and over-ventilation, show significantly more harm than those with a single successful intubation [33].

Bronchoscopy

Bronchoscopy has been used to visualize lung tissue and to examine the angle and branching of the bronchi within the lung. Changes in anatomy and angle can make accurate post-mortem and pre-mortem lung biopsies challenging [34]. Trans-nasal endoscopy has been shown to be successful in allowing detailed visual inspections of the respiratory and upper GI tracts. This has been particularly valuable in providing clues to cause of death. New surgical approaches to the nasopharynx using endoscopy have also been developed; using cadavers, the researchers discovered that endoscopic dissection of this region is feasible for treating nasopharyngeal carcinoma and the lesions can be removed completely [35]. This new surgical approach could not have been developed had it not been for the use of cadavers for exploring access to the cavities of sinuses in the head.

Laparoscopy

Gross anatomy demonstrations of laparoscopy have been implemented in several medical schools in the hope that exposure to such techniques would enhance learning and that minimally invasive surgical technology could be used to teach gross anatomy. If students could see the clinical relevance of abdominopelvic anatomy, traditional anatomical instruction would be reinforced. Laparoscopic demonstrations could also generate more interest in surgery as a field to pursue in medicine.

Laparoscopy has also been used on cadavers to reveal the tissues and makeup of structures such as the stomach wall, all four layers of which were visualized, their relative thicknesses measured, and uniformity across the organ identified [36]. Such exploration leads to a better understanding of human anatomy. It is a boon for clinicians and medical students alike as virtual reality dissections and virtual training become increasingly integral to learning as well as in providing exposure to new techniques.

Arthroscopy

Arthroscopy has been used to study joints and joint capsules in cadavers; to identify the structures that are at risk during procedures and to spot the neurovascular injuries that result from it [37]. Cadavers provide the most realistic mode of simulation for arthroscopic training. Both fresh-frozen and embalmed cadavers have been used in minimally invasive arthroscopic surgery training [38].

Otoscopy

Otoscopy has been used extensively to examine the middle ear, including resonance and air conduction [39]. In recent years, it has revolutionized facial nerve surgery, and practice of these techniques on cadavers though endoscopic exploration has allowed clinicians to determine that otoscopy can allow the tympanic facial nerve to be visualized, even including what were previously thought to be poorly-accessible regions such as the second genu and geniculate ganglion [40]. Additionally, it provides a promising new visualization technique for examining the middle and inner ear and has tremendous potential in both clinical and educational settings. Otoscopy provides morphological data for image diagnosis and oto-neurosurgery [41]. Endoscopy has allowed temporal bone anatomy and its relationship to the inner ear to be better understood.

Magnetic resonance imaging (MRI)

MRI provides unique insights into some tissues; the underlying pathogenesis and the histography of tissue and bones. This is especially apparent in images of the wrist [42]. MRI has also been used to study autonomic innervation and to map the nerves of the pelvis prior to surgery [43]. This has also led to better understanding of the anatomy of many muscles such as the soleus. A better understanding of the anatomy helps clinicians to diagnose injury to these muscles [44].

MRI can be not only expensive but also hard to schedule if there is no research-dedicated scanner. Fitting a cadaver into an already tight patient schedule can be more challenging than expected. MRIs can cost anywhere from 400 to 3,500 dollars per scan.

When researchers compared dissection of feet, specifically the Lisfranc joint, with MRI images, the MRI was more accurate and allowed for correct diagnosis of the Lisfranc joint instability, potentially leading to a more specific surgical management [45]. MRI is a highly accurate tool for diagnosing pathology and studying anatomy, but for cadavers, it is often cost and time prohibitive.

In a pediatric study in the UK, parents were more likely to consent to a minimally invasive or noninvasive autopsy of their deceased child to determine cause of death than an invasive one. The study showed that an MRI autopsy was as accurate as a conventional one. Within individual organ systems, postmortem MRI was most accurate for detecting cerebral, cardiac, and renal abnormalities, with the exception of ischemic brain injury and myocarditis [21].

Magnetic resonance angiography (MRA)

Often used in conjunction with MRI, MRAs have been useful for revealing anatomical variations in joints such as the shoulder. The anterior band of the inferior glenohumeral ligament is supposed to arise from the anteroinferior labrum, but we have observed that in some persons it originates from the anterior or anterosuperior labrum. There is a potential for diagnostic uncertainty and difficulties in such cadavers and patients. MRIs without an MRA of shoulders with such a variation can appear to be more serious than they are, including diagnosis of a labral tear or complete detachment [46].

Computed tomography angiography (CTA)

Computed tomography angiography (CTA) is an emerging tool; however, it is clear that postmortem CTAs have substantial value in forensic pathology. Postmortem CTA allows the coronary arteries to be displayed adequately in situ. Potentially fatal abnormalities such as severe stenosis of a coronary artery are well depicted with contrast [1]. Postmortem CTA allows the entire thoracic and abdominal aorta to be depicted, including branches such as the renal arteries, the celiac trunk, and the mesenteric arteries, which can enable aneurysms, ruptures, and dissections to be diagnosed [1].

CTA is superior to unenhanced postmortem CT in the depiction of traumatic vascular pathology. In addition, postmortem CTA brings added value in determining natural causes of death [1].

This is a minimally invasive procedure that allows vascular lesions to be diagnosed without disrupting the anatomical structures, which is inevitable during classic autopsy. Postmortem CTA, therefore, has the potential to prevent loss of physical evidence in a forensic investigation. Furthermore, it provides diagnostic information, without major destruction, from anatomical areas that are not well covered at classic autopsy (e.g., the craniocervical junction and the small pelvis). These areas are rarely dissected during classic autopsy without a specific suspicion of pathological conditions. Postmortem CTA, therefore, adds substantial value to classic forensic and non-forensic autopsies [1].

Elastography

This allows tissues to be assessed for stiffness. Clinicians are currently using cadavers to explore how elastography can be used to stage and diagnose liver failure, and as an alternative to liver biopsy. Additionally, surgical navigation systems for cancer have been tested on the prostate using elastography to distinguish cancerous from non-cancerous tissue; the feasibility of this method has been tested in cadavers [47]. Hatta, et al. applied shear wave elastography (SWE) to a cadaver with rotator cuff tears [48].

Tractography

Tractography has been used in cadavers to study neuronal fiber networks in the brain including the hippocampus structure and the “neuronal unit” concept, demonstrating to researchers that each neuronal unit consists of subicular hillock, dens, CA3, granular cell plates and folds, PL bars, and CA4 rods. This had previously been unconfirmed [49]. Diffusion imaging of postmortem brains can provide valuable data for diffusion tractography as a method for studying white matter pathways. The results of tractography in tracing neuronal pathways in large parts of the brain postmortem have shown it to be the preferred method of imaging. Tracts have been visualized and fibers estimated with greater success than with high-resolution MRI [50].

## Conclusions

After an extensive review of the literature, the authors have concluded that cadaveric imaging greatly advances our knowledge of the human body. Postmortem imaging is being used in a variety of ways around the world to better understand disease pathology, improve medical technology, and advance education and anatomical understanding. From our research, it appears that C-arm seems best applied in cadaveric studies in terms of its cost-effectiveness and efficiency. However, the use of advanced imaging techniques on cadavers is greatly improving what we know and impacting the quality of care that patients ultimately receive. The future of imaging in cadavers is full of possibility; however, space limitations, financial restrictions, and technical problems may be prohibitory in the use of advanced imaging techniques. The purpose of imaging techniques and the use of equipment differ largely among cadaveric laboratories.
